# MADS-Box Subfamily Gene *GmAP3* from *Glycine max* Regulates Early Flowering and Flower Development

**DOI:** 10.3390/ijms24032751

**Published:** 2023-02-01

**Authors:** Aijing Zhang, Haobo He, Yue Li, Lixue Wang, Yixuan Liu, Xinchao Luan, Jiaxin Wang, Huijing Liu, Shuying Liu, Jun Zhang, Dan Yao

**Affiliations:** 1 College of Life Sciences, Jilin Agricultural University, Changchun 130118, China; 2 College of Agronomy, Jilin Agricultural University, Changchun 130118, China

**Keywords:** *GmAP3*, MADS-box, soybean, tobacco, flower development

## Abstract

*AP3* has been studied and is reported to affect structural changes in floral organs in various plants. However, the function of the soybean *AP3* genes in flower development is unknown. Here, the full-length cDNA sequence of *GmAP3* was obtained by RACE and it was verified that it belongs to the MADS-box subfamily by a bioinformatics analysis. The expression of *GmAP3* is closely related to the expression of essential enzyme genes related to flower development. Yeast two-hybrid assays demonstrated that GmAP3 interacts with AP1 to determine the identity of flower organ development. A follow-up analysis showed that overexpression of the *GmAP3* gene advanced flowering time and resulted in changes in floral organ morphology. The average flowering time of overexpressed soybean and tobacco plants was 6–8 days earlier than that of wild-type plants, and the average flowering time of gene-edited soybean and tobacco plants was 6–11 days later than that of wild-type plants. In conclusion, *GmAP3* may directly or indirectly affect the flower development of soybean. The results of this study lay the foundation for further research on the biological functions of MADS transcriptional factors in soybeans.

## 1. Introduction

The MADS-box gene family is divided into type I and type II according to their different domains [1,2]. Type I MADS-box genes contain the *SRF* domain, and type II MADS-box genes contain the K region, which is less conserved. Type I genes are divided into Mα, Mβ, and Mγ types, according to different gene structures, while type II genes are divided into MIKCC and MIKC* types. Most MADS genes that have been reported are type II genes, such as floral homologs from the ABCDE model that belong to the MIKCC subfamily [3,4]. The formation of sepals, petals, stamens, carpels, and ovules is influenced by the interaction of these five types of genes. *AP1* is classified as a class A gene, and is a meristem and floral organ recognition gene that promotes the development of petals and sepals [5]. *PI* and *AP3* belong to class B genes in the MADS family. ap3 and pi mutants have identical phenotypes by producing the sepal structure and the carpel in Arabidopsis (*Arabidopsis thaliana*) [6]. *AG, SHP1, AGL1, SHP2,* and *STK* belong to C/D class genes. Class E genes include *SEP*, which can be further divided into four types, namely *SEP1, SEP2, SEP3,* and *SEP4*, which are involved in regulating Arabidopsis flower development [7].

The *GmAP3* gene belongs to class B in the MADS-box family. Class B genes have been reported in Arabidopsis, grapes (*Vitis vinifera*), lotuses (*Nelumbo nucifera*), tomatoes (*Solanum lycopersicum*), walnuts (*Juglans*), rice (*Oryza sativa* L.), cotton (*Gossypium* spp.), peanuts (*Arachis hypogaea* Linn.), apples (*Malus* spp.), cassavas (*Manihot esculenta Crantz*), and other plants [8,9,10,11,12,13]. Mutations in the class B genes lead to changes in the structure of floral organs and are also involved in the development of seeds [14]. In Arabidopsis, the class B gene *ABS* is expressed in the endothelial cell layer of mature ovules [15]. The Arabidopsis thaliana *PI* gene homolog *VvMADS9* in grapes is involved in forming petals and stamens. Results of in situ hybridization showed that *VvMADS9* was strongly expressed in stamens [16].

The MADS-box genes affect flowering time, flower development, seed development, and other processes, and have been shown to participate in the regulation of soybean (*Glycine max* (Linn.) Merr.) growth and development [17]. MADS-box genes regulate plant flowering time by responding to the external light environment and regulating endogenous signal changes. The expression of the *FT* gene is inhibited by the MADS-box family *FLC* homolog, GmFLC-like, resulting in delayed flowering [18]. In Arabidopsis, the overexpression of soybean *GmFULc* leads to early flowering, and the relative expression of flowering-related genes *FT*, *SOC1*, and *LFY* increases, which plays a role in promoting flowering [19]. MADS-box genes play an essential role in flower development and flower organ formation. A homologous MADS gene fragment has been cloned from the flower buds of *NJCMS2A*. This fragment had a high expression level in the soybean flower buds of the cytoplasmic male sterile line and a lower expression level in the soybean flower buds of the maintainer line. It was inferred that the significant expression of this gene may cause cytoplasmic male sterility in soybeans [20]. PCR and phenotypic identification of the *GmSVP1* transgenic tobacco-positive plants in T_0_ and T_1_ generations indicated that *GmSVP1* gene was involved in regulating flower development and flowering in soybean petals and stamens and other floral organs [21]. The MADS-box gene family is involved in the regulation of soybean seed development. Overexpression of the *GmFULa* gene increases the biomass accumulated during soybean growth without affecting plant height or changing flowering time and maturity, while promoting yield factors such as branch number, pod number, and 100-grain weight, thus increasing soybean yield [22].

Soybean is both an important food crop and oilseed crop. Regulation of soybean flowering time and flower organ morphology through genetic engineering and traditional breeding techniques has essential significance and broad application prospects to improve soybean quality and yield [23]. The *AP3* gene is involved in regulating plant flowering time, flower development, seed development, and other growth and development processes. Unfortunately, there are few *AP3* genes that have been identified so far in soybeans [24,25]. It is of great significance to clarify the action site, regulatory mechanism, and interaction mode of the *AP3* gene, and to improve the regulatory network of the soybean MADS-box gene for the study of soybean flower development.

## 2. Results

### 2.1. Gene Cloning and Sequence Analysis of GmAP3

To test if *GmAP3* is related to flower development in soybeans, total RNA was extracted from soybean flower buds (Figure S1). Using soybean bud cDNA as a template, we obtained the 3’-end sequence (711 bp) and 5’-end sequence (244 bp) by RACE (Figure S2). After sequence splicing, we successfully obtained the full-length cDNA sequence (923 bp) of *GmAP3* (Figure S3). Based on DNAMAN V6.0 multiple alignments, NCBI BLASTn alignment of the full-length cDNA sequence showed that *GmAP3* and the *GmNMH7* mRNA sequence of soybean MADS-box protein with a length of 948 bp in the Gen Bank database had the highest Max score, and the expected value was 0. The identities were 98% at best, but the differential gene is located on chromosome 18, while the target gene is located on chromosome 6, so this gene is presumed to be a new gene. The gene has been registered in Gen Bank with accession number Bank It1895068.

**Figure 1:**
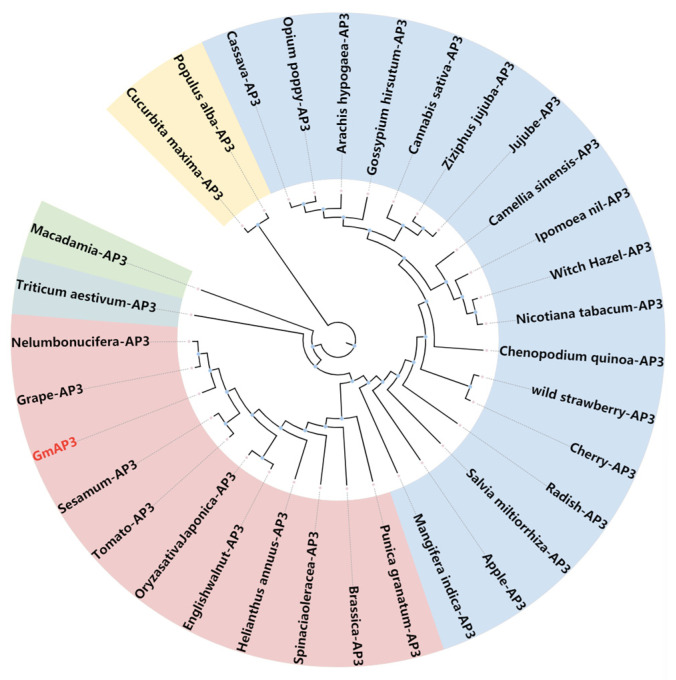
Phylogenetic analysis of *AP3* genes from Arabidopsis, grapes, lotuses, rice, tomatoes, cotton, peanuts, apples and cassavas. The phylogenetic tree was obtained through MEGA version 7.0 by Biomatters. Phylogenetic tree decorated by the ITOL website (https://itol.embl.de/; accessed on 15 December 2022). Protein sequences from the NCBI databases were used.

**Figure 2:**
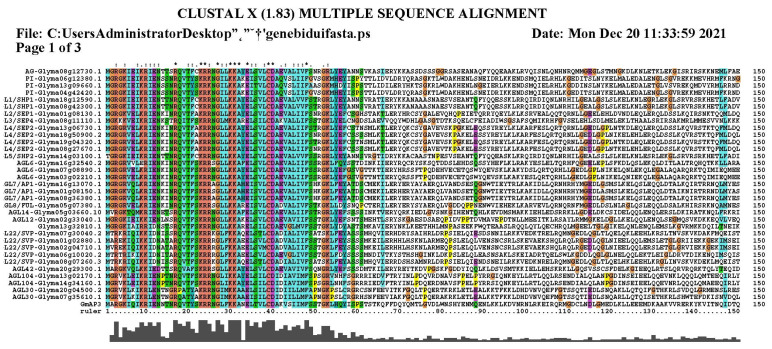
Comparative amino acid sequence analysis of 32 MADS-box gene families belonging to five categories of ABCDE based on MEGA7.0 and ClustalX. “*” indicates that all amino acid residues or nucleic acids in the aligned column are identical, “:” indicates that the column has a conservative substitution, “.” indicates that the column has a semi-conservative substitution.

To analyze the function of *GmAP3* in the MADS gene family, we downloaded the protein sequences of *AP3* genes from thirty-two different species including Arabidopsis, grapes, lotuses, rice, tomatoes, cotton, peanuts, apples, and cassavas from the NCBI database. Phylogenetic trees were constructed for homology comparison. The results showed that the *GmAP3* gene had high homology with the *AP3* gene in grapes and lotuses (Figure 1). Thirty-two protein sequences belonging to the five ABCDE categories of the MADS-box gene family in soybean were downloaded. The conservation of different MADS proteins in soybean was proved by amino acid sequence alignment with MEGA7.0 and ClustalX (Figure 2).

### 2.2. Genetic Transformation of GmAP3 Gene in Tobacco and Soybean

**Figure 3:**
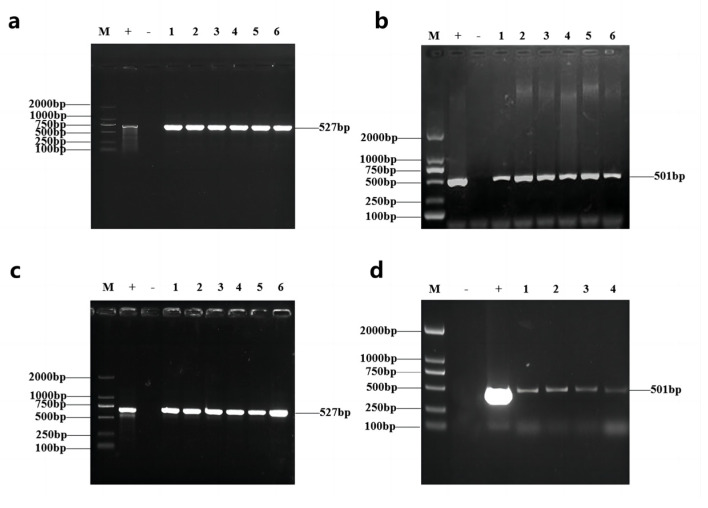
Identification of tobacco and soybean strains transgenic *GmAP3* gene. (**a**) PCR detection of some T_1_ overexpression transgenic tobacco, 1~6: sample; (**b**) PCR detection of some T_1_ editing transgenic tobacco, 1~6: sample; (**c**) PCR detection of some T_1_ overexpression transgenic soybean, 1~6: sample; (**d**) PCR detection of some T_1_ editing transgenic soybean, 1~4: sample. M: DL2000 DNA marker; +: positive plasmid; −: negative control.

We constructed the over-expression vector pCAMBIA3301-GmAP3 with pCAMBIA 3301 as the vector backbone (Figure S4). The editing vector, pBGK041Cas9-U6-GmAP3, was constructed with CRISPR/Cas BGK041 as the vector backbone (Figure S5). The recombinant plasmid plant expression vector was then transformed into A. *tumefaciens* EHA105. Six overexpression-positive tobacco plants, nine gene editing-positive tobacco plants, eleven overexpression-positive soybean plants, and fifteen gene editing-positive soybean plants were obtained after PCR detection of overexpression and editing vector-transformed plants with Bar and Cas9 as selection markers (Figure 3). Transgenic pCAMBIA3301-GmAP3-positive plants and pBGK041Cas9-U6-GmAP3-positive plants were harvested from the T_2_ generation, which were named OE-GmAP3 and Cas9-GmAP3, respectively.

### 2.3. Potential Expression Profiles of GmAP3 in Flowering Regulation

**Figure 4:**
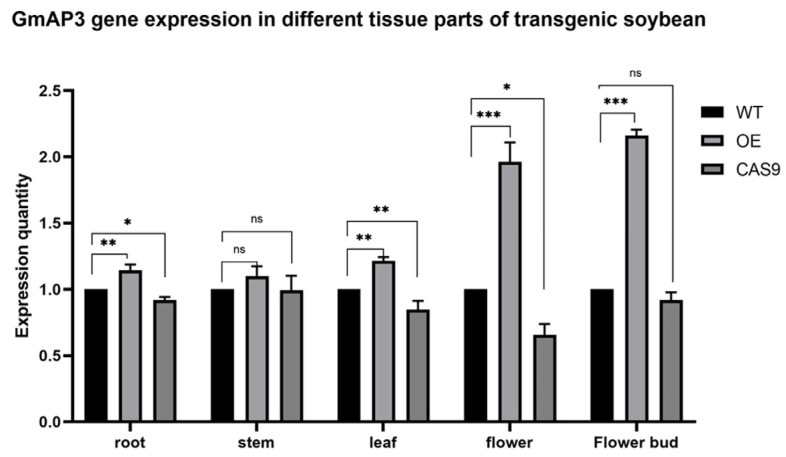
Expression pattern of *GmAP3* in different tissues or organs of transgenic soybean. Error bars represent the standard deviation. Significant differences according to Student’s *t*-test are indicated. *, **, and *** indicate significant differences at *p* < 0.05, *p* < 0.01, and *p* < 0.001 levels, respectively.

The expressions of the *GmAP3* gene were quantitatively evaluated in soybean roots, stems, leaves, flowers, and flower buds by real-time fluorescence quantitative PCR (Figure 4). The expression level of the *GmAP3* gene in flower buds and flowers of overexpressed strains was 2–3 times higher than that of the wild-type strains. There were no significant changes in the expression levels of the *GmAP3* in roots, stems, and leaves. The results suggest that *GmAP3* may be involved in flower bud and flower development in soybeans.

**Figure 5:**
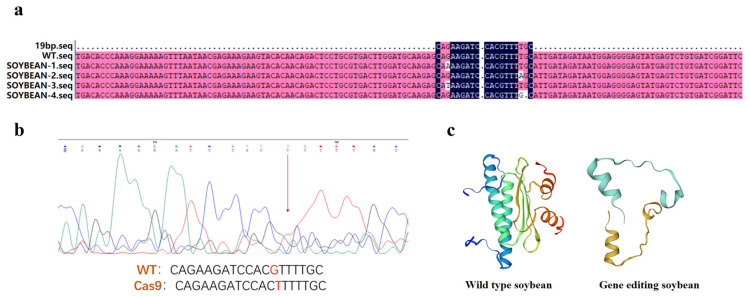
Mutation type and mutation efficiency analysis of gene-edited strains. (**a**) Partial sequence of mutation sites in transgenic soybeans; (**b**) peak map of target mutation in some plants; (**c**) prediction diagram of tertiary structure of some proteins.

The expression level of the *GmAP3* gene of edited strains was reduced. However, the changes in the expression levels of *GmAP3* in the gene-edited lines were less evident than in the overexpression lines. Subsequently, we analyzed the mutation type and mutation efficiency of the gene-edited strains. The results showed that 33 gene-edited plants were obtained from T_2_ soybean plants and 15 were mutated, with a mutation efficiency of 46%. The mutation types of transgenic soybean included pure and heterozygous mutations. Base substitution, base deletion, and base insertion occurred in the offspring of CRISPR/Cas9 editing. Among them, the proportion of base replacement was high, at about 60%. The results of amino acid sequence alignment and tertiary structure prediction showed that there was early termination of transcription in the base replacement mutant and the amino acid sequence of the mutant was significantly changed (Figure 5).

### 2.4. GmAP3 Regulated the Expression of Genes Involved in Flower Development

**Figure 6:**
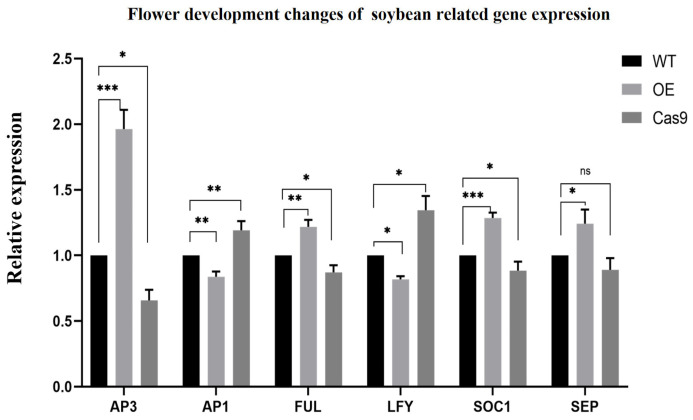
Expression analysis of key enzyme genes related to flower development in transgenic soybean. β-Actin was used as an internal control. Significant differences according to Student’s *t*-test are indicated. *, **, and *** indicate significant differences at *p* < 0.05, *p* < 0.01, and *p* < 0.001 levels, respectively. All data shown are means ± SD of three biological replicates.

To analyze the potential functions of the *GmAP3* in flower buds and flower development, we validated the expression of known genes critical for plant flower development by qRT-PCR. Total RNA was extracted from leaves of OE-GmAP3, Cas9-GmAP3, and control plants. We downloaded five known genes necessary for plant flower development from the NCBI database. *FUL* (*FRUITFUL*), *AP1* (*APETALA 1*), and *LFY* (*LEAFY*) are related to floral organ development. *SOC1* (*SUPPRESSOR OF OVEREXPRESSION OF CO 1*) is involved in regulating flowering time. SEP (*SEPALLATA*) is involved in many processes such as flower development and seed development. The expression levels of *FUL, SOC1,* and *SEP* genes were significantly higher in OE-GmAP3 plants than in the control, while the expression levels of *AP1* and *LFY* genes were lower than in the control. In Cas9-GmAP3 plants, the expression of *FUL* and *SOC1* genes decreased, the expression of *AP1* and *LFY* genes was higher than that of the management, and the manifestation of the *SEP* gene did not change significantly (Figure 6). These results indicated that *GmAP3* could directly or indirectly participate in flower and seed development pathways in soybean by modulating the expression of genes that are known to be critical for flower development.

### 2.5. GmAP3 Affected Flower Development by Regulating AP1

**Figure 7:**
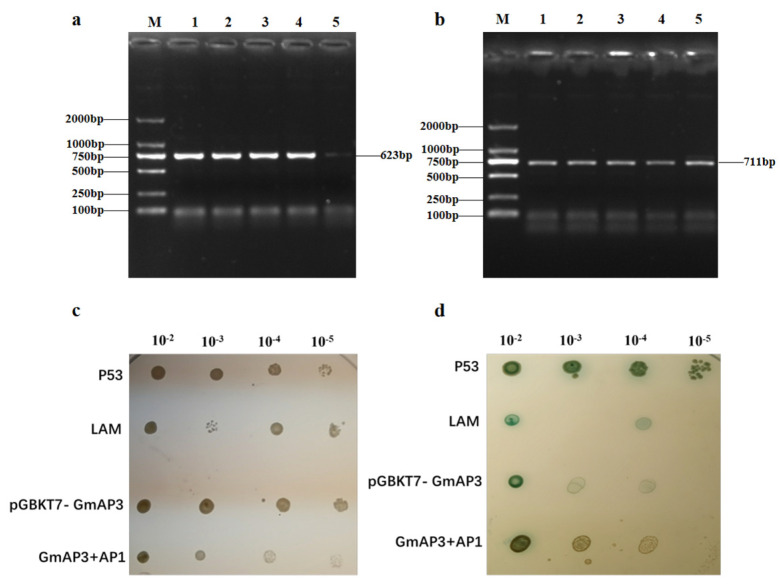
Interactions between *GmAP3* and *AP1* in yeast. M:DGL2000 DNA, 1~5: sample. (**a**) pGBKT7-GmAP3 vector; (**b**) pGADT7-AP1 vector; (**c**) SD-leu-Trp culture medium; (**d**) SD-Leu-His-Ade-Trp culture medium.

Studies have shown that the formation of petals and stamens is regulated by the joint action of B (*AP3*, *DEF*, *P*, and *GLO*) and A (*AP1*, *AP2*, *AG*, and *FUL*) genes of the MADS family. In a previous study, we analyzed the promoter sequence of *GmAP3* and found that the promoter sequence of the *GmAP3* gene contained a binding site to *AP1*. In the present study, the expressions of *AP1* were significantly decreased in OE-GmAP3 and the expressions of *AP1* were increased considerably in Cas9-GmAP3 soybean. To explore the possible roles of *GmAP3* and *AP1* in flower development, the bait vector pGBKT7-GmAP3 and the prey vector pGADT7-AP1 were constructed successfully by ligating the *AP1* plasmid with the digested product of the pGADT7 vector plasmid (Figure 7a,b). Subsequently, pGADT7-T was introduced into the competent cell of Saccharomyces cerevisiae AH109 in combination with pGBKT7-GmAP3. After 5d, it was found that the bacteria could grow normally on SD-Trp, indicating that pGBKT7-GmAP3 was not toxic. pGADT7-AP1 was introduced into the competent cell of Saccharomyces cerevisiae AH109 in combination with pGBKT7-GmAP3; the yeast cells co-transformed could grow on the SD-leu-Trp and SD-Leu-His-Ade-Trp in X-α-Gal medium (Figure 7c,d). The results showed that *GmAP3* could interact with *AP1* proteins.

### 2.6. GmAP3 Advanced the Flowering Time

**Figure 8:**
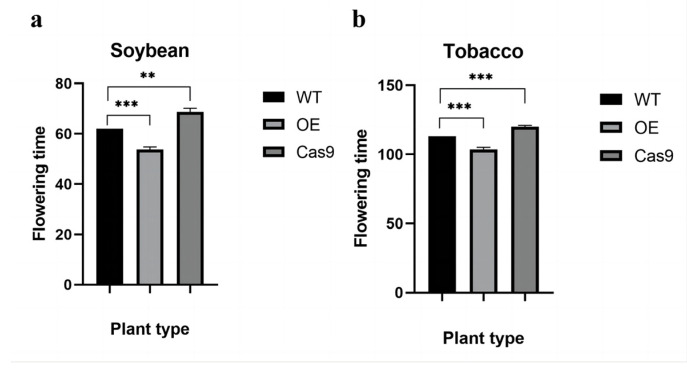
Comparison of flowering time between T2 generation of positive transgenic soybean plants (**a**), T2 generation of positive transgenic tobacco plants (**b**) and wild-type plants.Significant differences according to Student’s *t*-test are indicated. ** and *** indicate significant differences at *p* < 0.01 and *p* < 0.001 levels, respectively. All data shown are means ± SD of three biological replicates.

To further investigate the function of *GmAP3*, we observed the flowering pattern of transgenic plants. Surprisingly, the average flowering time of OE-GmAP3 soybean was 8 d earlier than that of the wild-type, and that of Cas9-GmAP3 soybean was 11 d later than that of the wild-type (Figure 8a). The above results were significantly different from the average flowering time of control wild-type soybean plants, which was 62 d. Flowering time changes recorded in transgenic tobacco were observed in artificial climate chambers. It was found that the average flowering time of OE-GmAP3 tobacco was 7 d earlier than that of the wild-type. Additionally, that of Cas-AP3 tobacco was 8 d later than the wild-type (Figure 8b), which were significantly different from the average flowering time of 113 d of control wild-type tobacco plants. The results indicate that the *GmAP3* gene may regulate the flowering time changes in soybean.

### 2.7. GmAP3 Advanced Flower Organs Development

**Figure 9:**
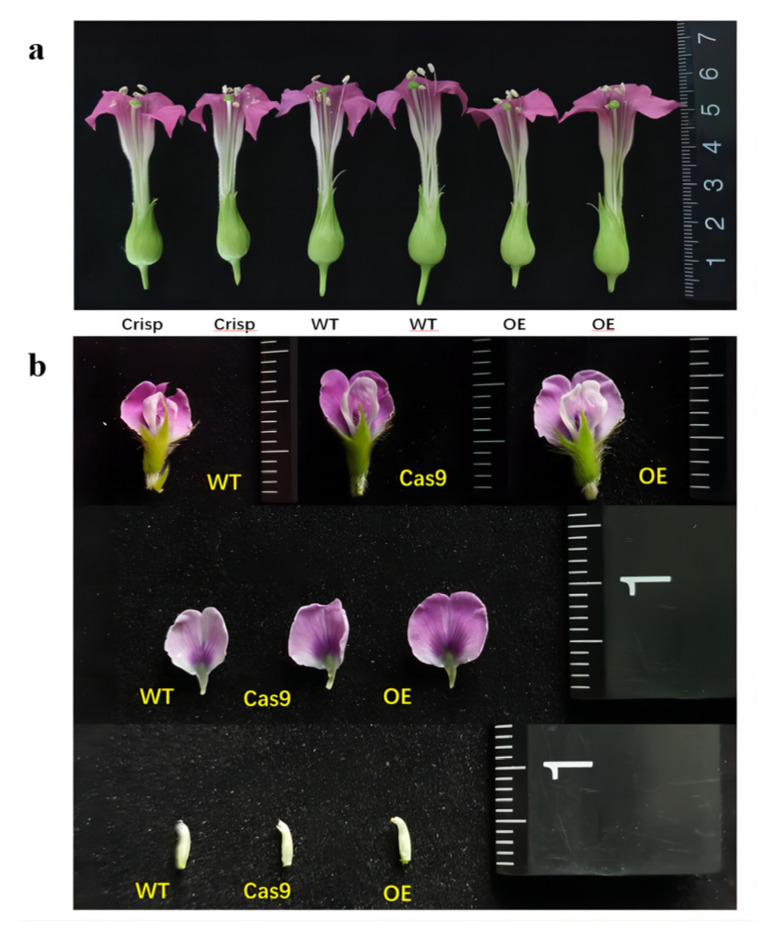
Phenotypic analysis of *GmAP3* transgenic plants. The score value of the scale ruler is 1 mm. (**a**) Morphological map of floral organs in transgenic and wild-type tobacco; (**b**) comparison of flower organs between transgenic and wild-type soybean.

The *AP3* gene is associated with petal formation in Brassica, and it may be involved in regulating stamen development in plants. In this research, we selected three blooming flowers from each of OE-GmAP3 tobacco, Cas9-GmAP3 tobacco, and wild-type plants, and measured and recorded the sizes of petals, stamens, and stigmas. The observation results showed that the stamens of OE-GmAP3 tobacco were shorter than those of the wild-type, while the stamen length of Cas9-GmAP3 plants did not change significantly (Figure 9a).

**Figure 10:**
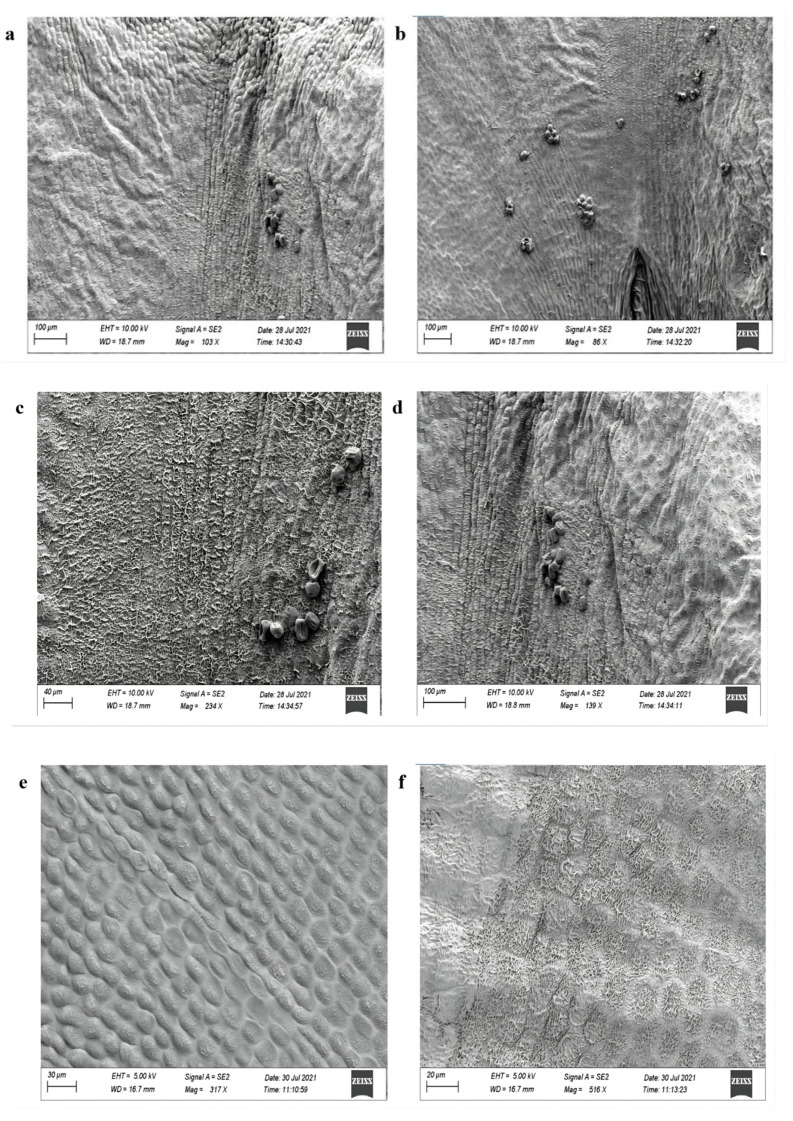
Comparison of scanning electron microscope results of petals between transgenic plants and wild-type plants. (**a**,**c**,**e**) OE-GmAP3 soybean; (**b**,**d**,**f**) wild-type plant.

**Figure 11:**
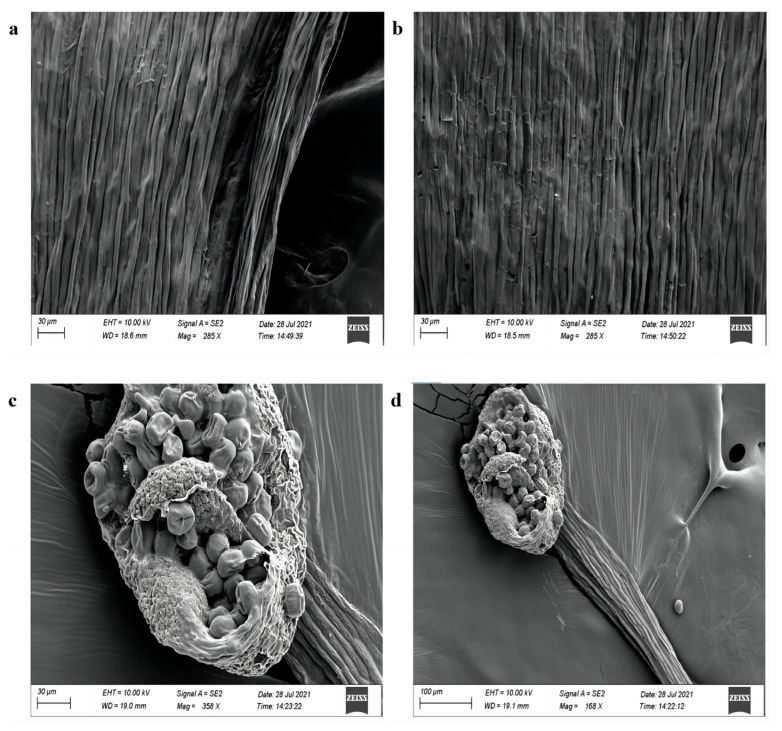
Comparison of scanning electron microscope results of pistils between transgenic plants and wild-type plants. (**a**) Pistil of wild-type soybean; (**b**) pistil l of OE-GmAP3 soybean; (**c**) pistil of wild-type soybean; (**d**) pistil of OE-GmAP3 soybean.

We measured and compared the shape and length of petals, sepals, pistils, and other major flower-related indicators of wild-type, OE-GmAP3 soybean, and Cas9-GmAP3 soybean at full bloom. The results show that the diameter of the flag petals of the overexpressed soybean plants is more significant than that of the wild-type (Figure 9b). The cell morphological changes in the transgenic plants were investigated in more detail by scanning electron microscopy (SEM). SEM images of soybean flowers revealed that, compared with the wild-type, the adaxial cell structure of flag petals is more compact and dense, the cell shape is plumper, and the difference is more evident in OE-GmAP3 soybean (Figure 10 and Figure 11).

### 2.8. Overexpression of GmAP3 Increased Seed Yield

Overexpression of *SOC1*, by the MADS-box type II transcription factor, impacts the total seed yield in transgenics, and the expression of *OsMADS87* regulates seed size in rice [26,27]. To investigate the role of *GmAP3* in seed yield, we selected five plants from each positive transgenic soybean line randomly. We measured the statistics of the main agronomic characters, such as the number of branches, the number of primary stem nodes, the number of three pods, the number of four pods, the number of whole pods, and the weight of 100 seeds. Surprisingly, there were significant differences in the number of four pods, plant height, and the total number of pods between the OE-GmAP3 and Cas9-GmAP3 soybean compared with the wild-type. The total number of pods in the progeny of soybeans overexpressing the *GmAP3* gene in the T_2_ generation increased, indicating that overexpression of the *GmAP3* gene may improve seed yield (Table 1).

## 3. Discussion

AP3-like genes have been reported in various plants, and *AP3* genes play an essential role in plant flower development. The *AP3* gene is expressed in petals and stamens in loquat, and when introduced into Arabidopsis, the petals become narrower [28]. The *AP3* gene of buckwheat was overexpressed to turn long stamens into filamentous stamens in Arabidopsis [29]. In this study, the stamens of the overexpressed plants were shorter than those of the wild-type when the flower organs of transgenic tobacco matured, indicating that *AP3* may be involved in regulating stamen development in plants. *AP3*, as a class B gene of the “ABC” model, may work with class A genes (*AP2*, *AG*, and *FUL*), class C genes (*AG*, *STK*, and *AGL11*), or other genes to control petal and stamen growth [30]. Unusual flower morphology appeared in *A. caudigerum*. This may be because that subfunctionalization may have contributed to persistent functional *AP3* paralogs, i.e., the copies may have become pseudogenes [31]. In Orchids (*Oncidium Gower Ramsey*), the OMADS8 *PI* motif and C-terminus and the interaction of OMADS8 with *AP3* orthologs form higher-order heterotetrameric complexes with implications for regulating petal/stamen formation in orchids and transgenic Arabidopsis [32]. Nevertheless, until now, little research has investigated the interaction of *AP3* with other genes involved in soybean stamen development. This indicates that there is still considerable room to study the regulatory network mechanism of *AP3* in soybean stamens.

Plant MADS-box genes have been extensively studied by CRISPR/Cas9 technology. The petal and stamen properties of Arabidopsis can be controlled by the strawberry AP3 gene. The function of *AP3* in strawberry flower development can be characterized using the CRISPR/Cas9 genome editing system [33]. *SlMADS1* acts as a negative regulator of fruit ripening. To investigate the biological function of *SlMADS1*, KO-SlMADS1 (knock-out) tomato mutants were generated by CRISPR/Cas9 technology and over-expression of SlMADS1 (OE-SlMADS1) [34]. In this work, we constructed a CRISPR/Cas9 editing vector. We found that the effect of *AP3* in soybean flower development was not apparent in CRISPR/Cas9 transgenic plants compared with overexpressing transgenic plants. In this experiment, the editing efficiency of the CRISPR/Cas9 system is different in soybean and tobacco. The editing efficiency is lower in soybean and the off-target rate is higher. The reason for the low editing efficiency may be that the soybean CRISPR/Cas9 system still needs to be improved. The mismatch between the sgRNA sequence and the PAM sequence binding region, the difference in the off-target results caused by different sequences of PAM, the tolerance of Cas9 nuclease to mismatched bases the number and position of mismatched bases and different distributions, etc., may lead to differences in editing efficiency of the CRISPR/Cas9 system [35,36,37,38]. Conducting multi-target analysis of gene-edited plants, perfecting the CRISPR/Cas9 system, and improving the genetic transformation method will help to further explore the function of the MADS gene family in crops with a complex genome, such as soybean [39,40,41,42].

MADS-box genes of the *AP3* type are key regulators of petal initiation and development [43]. Genes encoding MADS-domain proteins have been targets for selection during crop domestication [44]. The *AP3* gene not only regulates flower development by itself, but also cooperates with other factors to participate in the complex regulatory network of flower development. *AP3* was co-expressed with GAIP-B-like and *YABBY5* (*YAB5*), which regulated the development of the petal, stamen, and nectar in *Viola prionantha* [45]. *ZaMADS70* (SEP3-like) interacted with *ZaMADS48* (AP3-like), facilitating the loss of petals in *Z. armatum* [46]. In addition, *CsFLC1* controls flowering time, possibly by regulating *AP3* in tea plants [47]. Additionally, AP3-like proteins could form heterodimers with *CcMADS20*, which is involved in the regulation of petal and stamen development during the evolutionary process of citrus plants [48]. In order to explore the regulation pathway of *GmAP3* on flowering in soybean, we analyzed the expression levels of known genes critical to flower development in OE-GmAP3 and Cas9-GmAP3 plants. We found that the expression of *GmAP3* affected the expression levels of *FUL*, *SOC1*, *SEP*, *AP1*, and *LFY* genes. The interaction between *GmAP3* and *AP1* was further explored by a yeast two-hybrid experiment. In future studies, we will conduct more experiments to verify this result. Furthermore, overexpression of *GhKTI12* enhanced seed yield and biomass yield in *Nicotiana Tabacum*, and the expression of the *AP3* gene was changed in *GhKTI12* overexpressing plants [49]. *AP3* can regulate the transcriptional activity of cell wall invertase which regulates reproductive development in *Arabidopsis thaliana* [50]. In our study, the total number of pods of OE-GmAP3 increased. These results indicate that *GmAP3* may be an indirect regulator of seed development, which is an interesting topic for future investigations.

## 4. Materials and Methods

### 4.1. Plant Materials

The materials used in this research were the four-grain pod mutants screened at the Biotechnology Center of Jilin Agricultural University in 2008. The material was mutated by the soybean variety “JN18” (Ji Shen bean 2006), and the quadruped ratio was on average 20% higher than the wild-type material. The yield of the mutant cell was about 15% higher than the wild-type material. The mutant material has been maintained since 2008 and has been stable after field identification and screening for many years. Tobacco “NC89” wild-type seeds were preserved in the laboratory of the Oil Crops and Molecular Breeding Team of Jilin Agricultural University.

### 4.2. RNA Isolation and Reverse Transcription

RNA was extracted using an RNApure Plant Kit (CoWin Biosciences, CW0559). The quality of RNA was detected by agarose gel electrophoresis and NANO DROP ONE (Thermo Scientific, Shanghai, China). Reverse transcription was performed using HiFiScript gDNA Removal cDNA Synthesis Kit (CoWin Biosciences, CW2582).

### 4.3. Cloning of Full-Length GmAP3 cDNA

Gene-specific primers were designed using Primer 7.0 (Table S1). The partially known sequence of the soybean *GmAP3* gene was obtained from the SoyBace website (Gly-ma18g33910.1; https://www.soybase.org/ (accessed on 11 November 2015). An SMARTer RACE 5′/3′ Kit (Takara, 634858) was employed to obtain the full-length cDNA for *GmAP3*. The purified PCR products were sub-cloned into PMD18-T (Takara, 6011).

### 4.4. Bioinformatics Analysis

The secondary structure prediction was obtained at https://npsa-prabi.ibcp.fr/ (accessed on 22 November 2021). The tertiary structure prediction was obtained at http://swissmodel.expasy.org/ (accessed on 22 November 2021). The ITOL phylogenetic tree beautification tool can be found at https://itol.embl.de/ (accessed on 15 December 2022).

### 4.5. Construction of Overexpression and Editing Vectors

To construct the overexpression vector, the pCAMBIA3301 empty vector was linearized using the restriction enzymes *BamH*I and *Spe*I. The linearized fragments were recovered with a Gel Extraction Kit (CoWin Biosciences, CW2302). The recovered product was ligated with *GmAP3* by T4 DNA Ligase (TaKaRa, 2011A). We constructed an editing vector with the CRISPR/CasBGK041 (BIOGLE Hangzhou) vector as the backbone.

### 4.6. Transformation of Soybean by Pollen Tube Pathway

We sowed the wild-type soybean variety “JN18” in the transgenic cultivation base of Jilin Agricultural University in May; extracted many recombinant plasmids, pCAMBIA3301-GmAP3, and pBGK041Cas9-U6-GmAP3; and transformed soybeans by the pollen tube channel method in July. According to the growth characteristics of soybean flowers, after the flowers were pollinated, 5 µL of the recombinant plasmid was extracted with a pipette and injected into the pollen tube channel formed during the flowering and fertilization of soybean flowers.

### 4.7. Ectopic Expression in Tobacco

The *GmAP3* sequence was cloned into the plant binary vector. The recombinant plasmids were used for tobacco transformation via the leaf disk transformation method.

### 4.8. Quantitative Real-Time PCR Analysis

The melting curve was determined to confirm the specificity of the amplified fragment. Actin genes were used as reference genes to normalize the expression data. The relative expression of genes was calculated according to the 2^−ΔΔCT^ method. All experiments were repeated three times independently.

### 4.9. Electron Microscopy Analysis

Cell morphological changes observed in OE-GmAP3, Cas9-GmAP3, and wild-type flowers were analyzed by scanning electron microscopy. The model of the electron microscope was a Zeiss Gemini Sigma 300 VP SEM.

### 4.10. Yeast Two-Hybrid Assays

The full-length cDNA *GmAP3* was ligated into pGBKT7 bait vector after amplification with the primers carrying restriction sites of *EcoR*Ⅰ and *BamH*Ⅰ, and the full-length cDNA *AP1* was ligated into pGADT7 bait vector after amplification with the primers carrying restriction sites of *EcoR*Ⅰ and *Xho*Ⅰ. pGADT7-AP1 and pGBKT7-GmAP3 were introduced into the competent cell of Saccharomyces cerevisiae AH109. Then, the bacterial solution was taken, coated on SD-leu-Trp medium and SD-Leu-His-Ade Trp medium, and cultured at 30 °C for five days in a constant temperature incubator. An amount of 5 µL of X-α-Gal working solution was added to the medium requiring X-α-Gal color verification, and the color development was observed after 30 min of culture at 30 °C.

## 5. Conclusions

In summary, we obtained *GmAP3* overexpressed lines and gene-edited lines through the genetic transformation of soybean and tobacco. We found that the *GmAP3* gene is closely related to essential enzyme genes involved in flower development and seed development, such as *FUL*, *SOC1*, *SEP*, *AP1,* and *LFY*. Research confirmed the interaction between the *GmAP3* protein and the *AP1* floral organ recognition protein by a yeast two-hybrid assay. When we observed the phenotype of transgenic plants, we found that overexpression of the *GmAP3* gene advanced the flowering time of plants and led to changes in floral organ morphology. Our study may aid in the understanding of the molecular basis of agronomically important traits of soybean, such as flower development, seed development, and other physiological processes.

## Figures and Tables

**Table 1:** Determination of agronomic characters of transgenic soybean. Different lowercase letters represent significant differences from each other, and repeated letters represent no significant differences.

Strain	Number of Branches	Number of Nodes on the Main Stem	Number of Four-Seed Pods	100-Grain Weight/g	Height/cm	Number of Pods Per Plant
JN18CK	7.00 ± 1.00 ^a^	18.67 ± 1.53 ^ab^	1.33 ± 0.58 ^b^	18.83 ± 0.29 ^a^	67.83 ± 1.61 ^ab^	97.67 ± 1.15 ^bc^
Cas9-GmAP3-26	6.33 ± 0.58 ^a^	16.00 ± 2.65 ^b^	1.33 ± 0.58 ^b^	17.33 ± 2.08 ^a^	67.33 ± 3.79 ^ab^	114.33 ± 24.54 ^abc^
Cas9-GmAP3-30	4.00 ± 0.00 ^a^	17.00 ± 1.00 ^ab^	1.33 ± 1.15 ^b^	17.33 ± 1.15 ^a^	68.5 ± 5.50 ^ab^	68.33 ± 15.95 ^c^
Cas9-GmAP3-46	6.33 ± 1.15 ^a^	17.33 ± 0.58 ^ab^	2.33 ± 2.08 ^ab^	18.33 ± 1.15 ^a^	64.07 ± 5.25 ^bc^	106.00 ± 12.29 ^abc^
Cas9-GmAP3-56	4.33 ± 1.15 ^a^	17.00 ± 1.00 ^ab^	0.67 ± 1.15 ^b^	16.67 ± 0.58 ^a^	67.33 ± 3.51 ^c^	98.00 ± 43.59 ^bc^
Cas9-GmAP3-67	5.67 ± 1.15 ^a^	19.00 ± 1.00 ^ab^	0.33 ± 0.58 ^b^	18.67 ± 1.15 ^a^	65.33 ± 2.52 ^abc^	95.00 ± 22.65 ^bc^
OE-GmAP3-41	5.33 ± 1.15 ^a^	17.00 ± 3.00 ^ab^	2.00 ± 1.00 ^b^	17.33 ± 0.58 ^a^	66.83 ± 4.54 ^ab^	107.33 ± 67.95 ^abc^
OE-GmAP3-30	5.67 ± 1.53 ^a^	17.33 ± 1.53 ^ab^	1.00 ± 1.00 ^b^	16.67 ± 1.53 ^a^	68.33 ± 7.51 ^ab^	115.00 ± 25.16 ^abc^
OE-GmAP3-61	6.33 ± 1.15 ^a^	19.67 ± 0.58 ^a^	5.00 ± 2.65 ^ab^	18.00 ± 1.00 ^a^	73.83 ± 3.62 ^a^	164.00 ± 32.23 ^ab^
OE-GmAP3-74	4.33 ± 0.58 ^a^	18.33 ± 1.15 ^ab^	3.33 ± 2.08 ^ab^	18.33 ± 1.15 ^a^	72.67 ± 3.79 ^ab^	84.67 ± 7.23 ^bc^
OE-GmAP3-82	6.33 ± 3.51 ^a^	18.67 ± 0.58 ^ab^	7.33 ± 6.51 ^a^	18.33 ± 4.04 ^a^	69.33 ± 3.06 ^ab^	193.33 ± 94.63 ^a^

## Data Availability

Not applicable.

## Supplementary Materials

The following supporting information can be downloaded at: https://www.mdpi.com/article/10.3390/ijms24032751/s1.
