# Supplementary material for: MADS-Box Subfamily Gene GmAP3 from Glycine max Regulates Early Flowering and Flower Development
Source: Int J Mol Sci. 2023 Feb 1;24(3):2751. doi: 10.3390/ijms24032751 (PMC9917172; doi:10.3390/ijms24032751)
Supplement: Supplementary file 1 [file ijms-24-02751-s001.zip › Table S1 (All Primers).pdf]

Table S1. List of all the primers.

Experiment	Primer	Sequence
Gene cloning	*GmAP3* sense primer	CCGGAATTCATGGCTAGAGGAAA
	GmAP3 antisense primer	CGCGGATCCGAAAGTAAAGGGT
Verification of positive plant materials	Bar sense primer	CACCATCGTCAACCACTACATCG
	Bar antisense primer	TGAAGTCCAGCTGCCAGAAAC
Internal reference gene for qRT-PCR	*ACTIN* sense primer	CGGTGGTTCTATCTTGGCATC
	ACTIN antisense primer	GTCTTTCGCTTCAATAACCCTA
Expression of key genes critical for plant flower development	*GmAP3*q sense primer	AACCAAGCCATCCTAGTGCC
	*GmAP3*q antisense primer	CGCGTTCACTTGTCATCGTC
	FUL sense primer	CCCCACAAGCAAGATGGA
	FUL antisense primer	AATTGTCAGGACTCCGGTGG
	AP1 sense primer	ATGTTCAAAAGTGGCTGCGG
	AP1 antisense primer	AGCAAGCACAATGGGAGCAT
	FLY sense primer	GTTACTGGGACGCAGGTCAA
	FLY antisense primer	CCTCCGTTGCCGTTATCAT
	SOC1 sense primer	ATGGGAAGCTGGTGAGCAAA
	SOC1 antisense primer	TTGCACCCAAAGGCTGGAA
	SEP sense primer	AGCTACCGTGAGTACTTGAGC
	SEP antisense primer	GAGATGAATCCAGTTGGCGCT
Yeast two-hybrid assays	pGBKT7-AP3 sense primer	CCGGAATTCATGGCTAGAGGAAA
	pGBKT7-AP3 antisense primer	CGCGGATCCGAAAAGTAAAGGGT
	pGBKT7-AP1 sense primer	CCGGAATTCCGGTTTTTTTTTTTTT TTTTTTTTTTT
	pGBKT7-AP1 antisense primer	CGGATCCGCGATGATTCCGAGTCA CAGGGAAAT
