# Supplementary figures and images for: MADS-Box Subfamily Gene GmAP3 from Glycine max Regulates Early Flowering and Flower Development
Source: Int J Mol Sci. 2023 Feb 1;24(3):2751. doi: 10.3390/ijms24032751 (PMC9917172; doi:10.3390/ijms24032751)

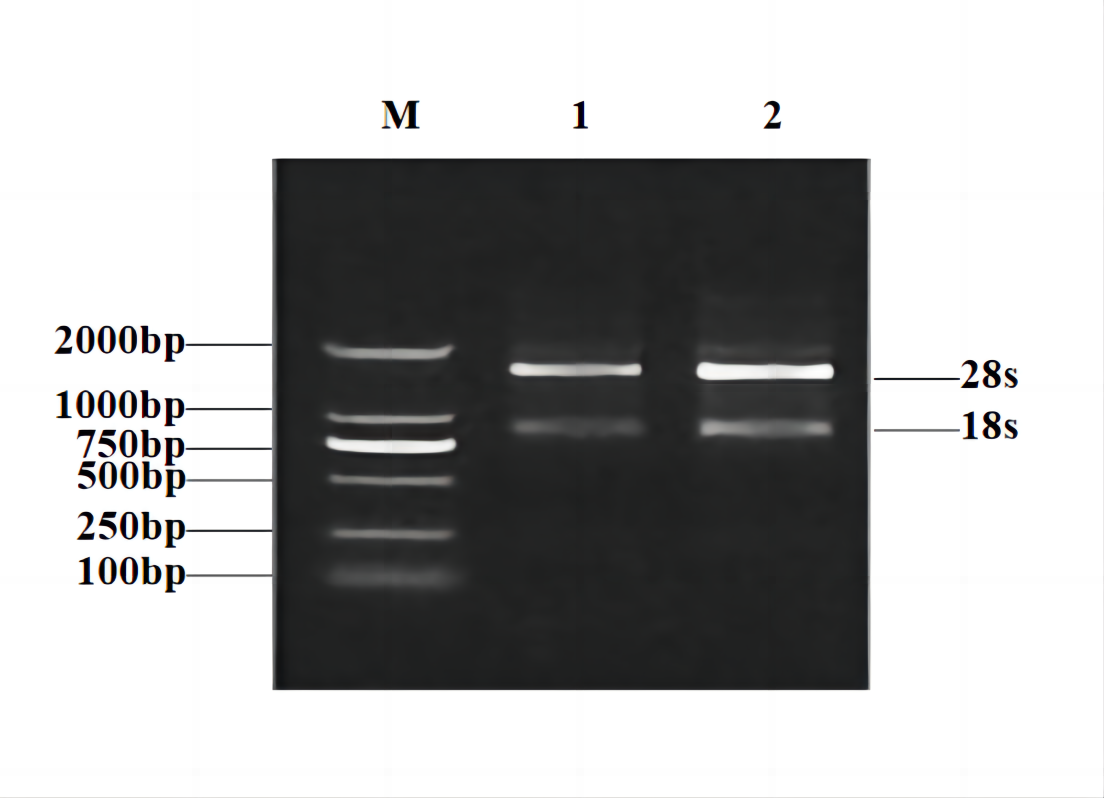

Supplement: Supplementary file 1 [file ijms-24-02751-s001.zip › Figure S1.png]

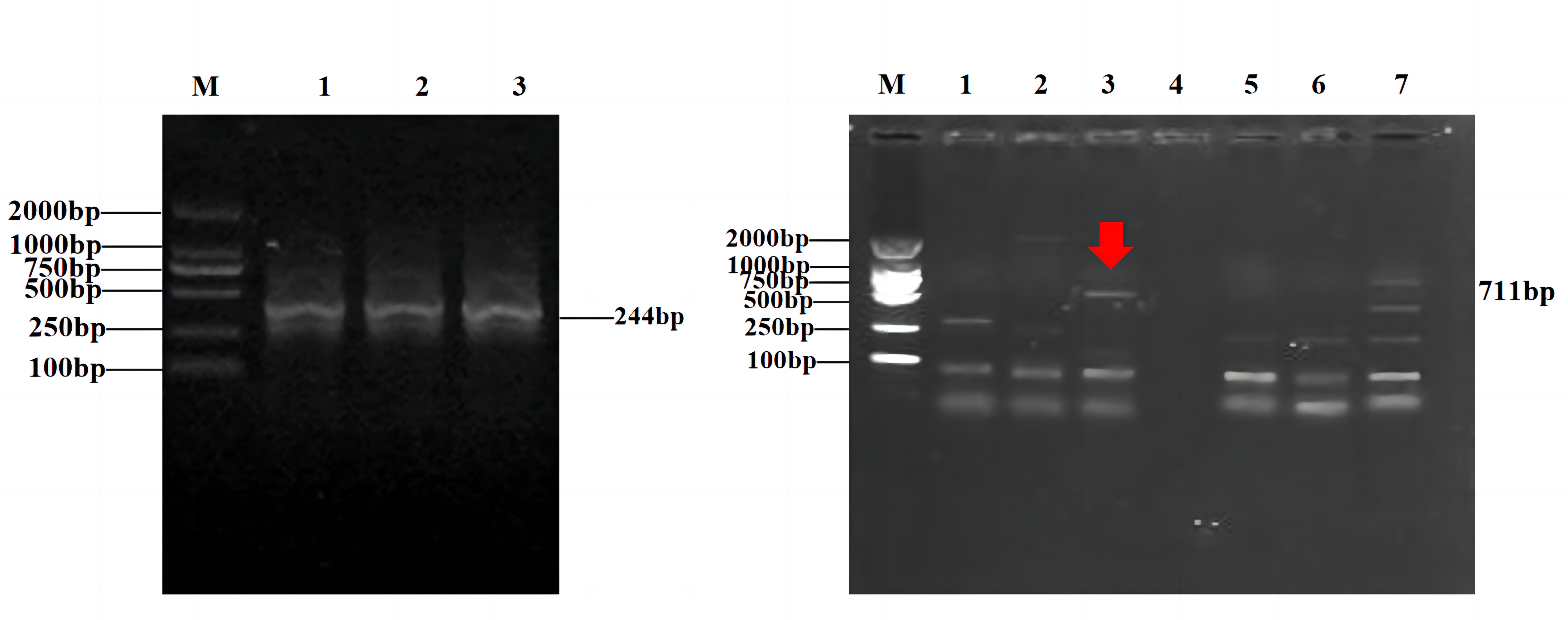

Supplement: Supplementary file 1 [file ijms-24-02751-s001.zip › Figure S2.png]

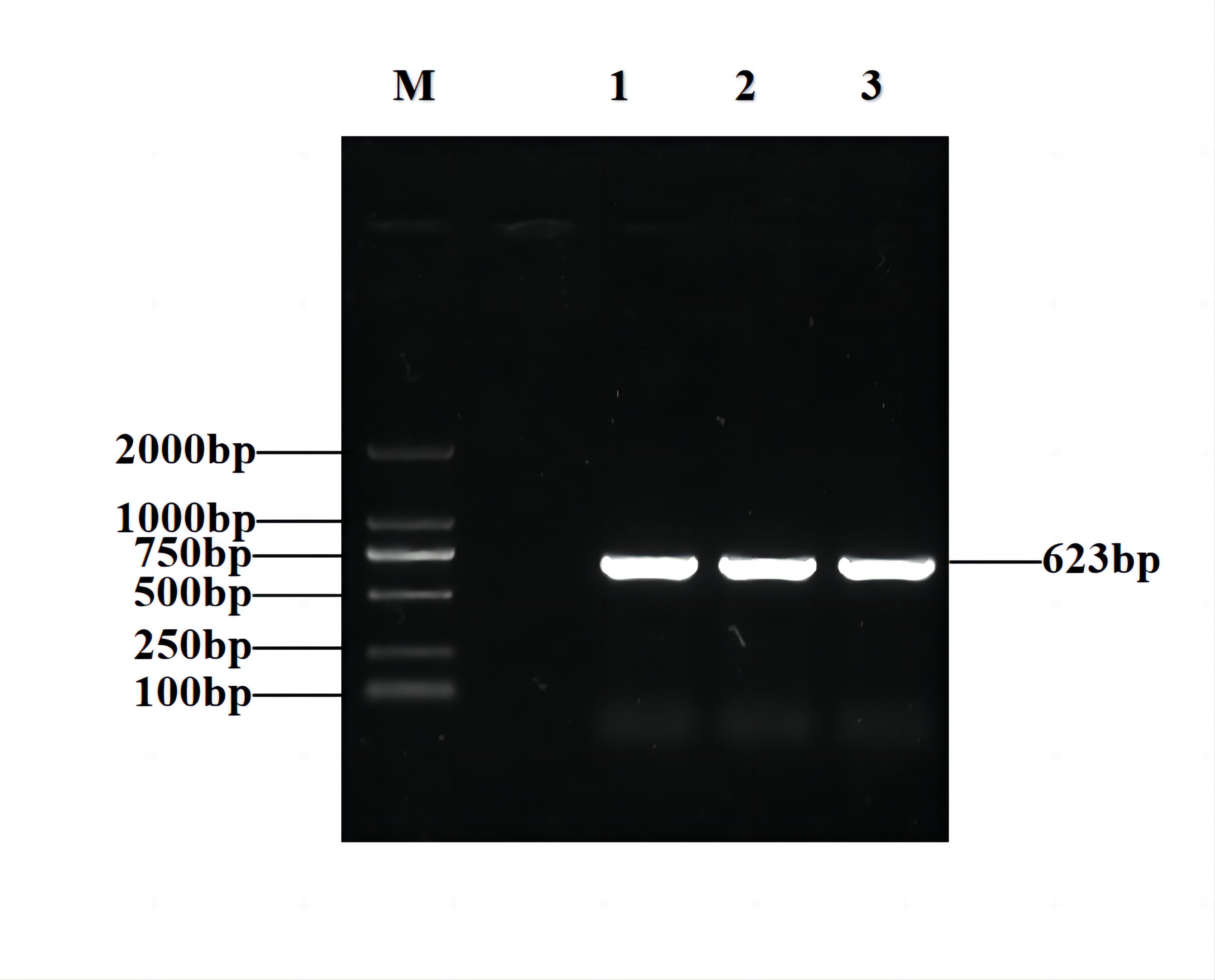

Supplement: Supplementary file 1 [file ijms-24-02751-s001.zip › Figure S3.png]

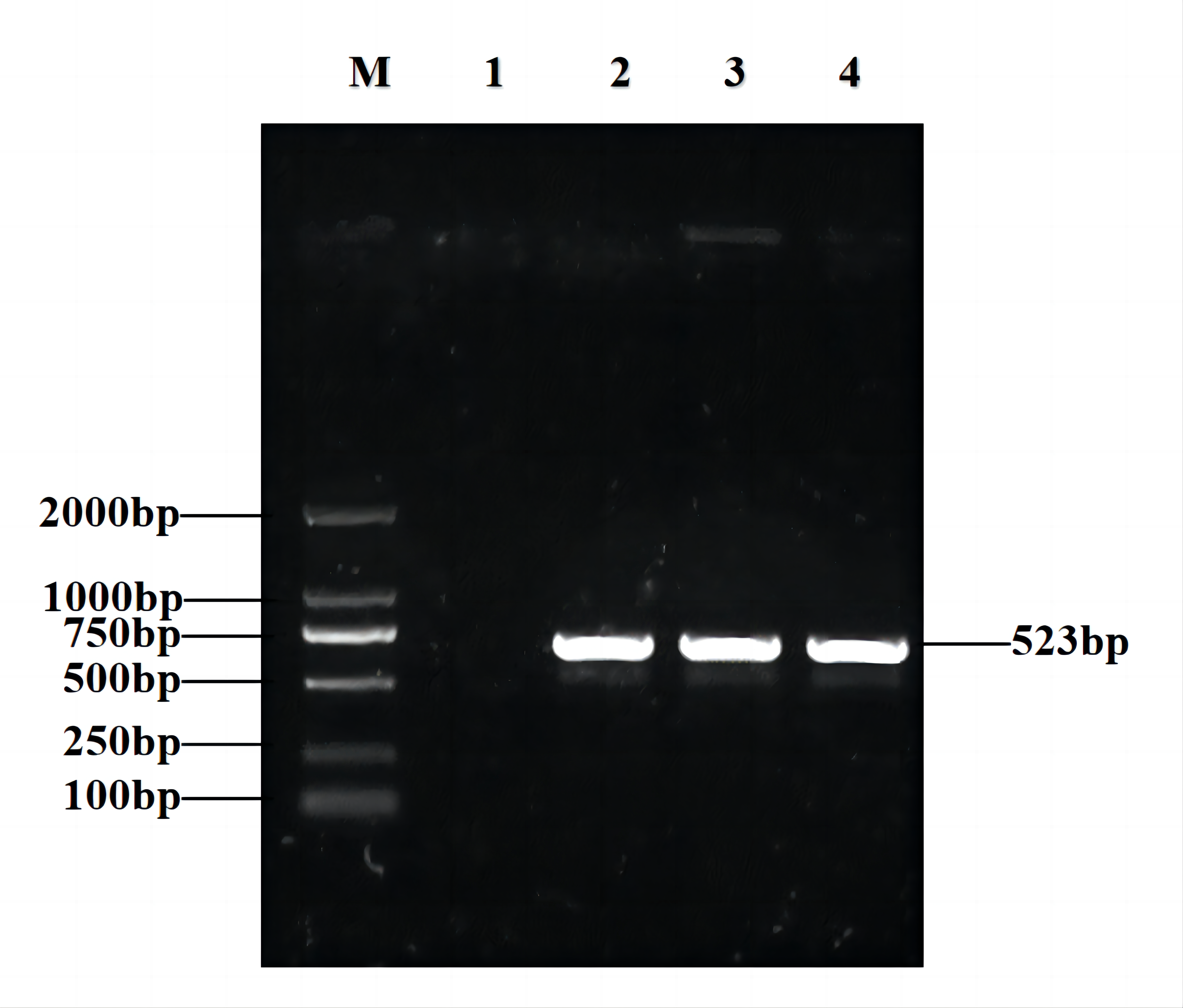

Supplement: Supplementary file 1 [file ijms-24-02751-s001.zip › Figure S4.png]

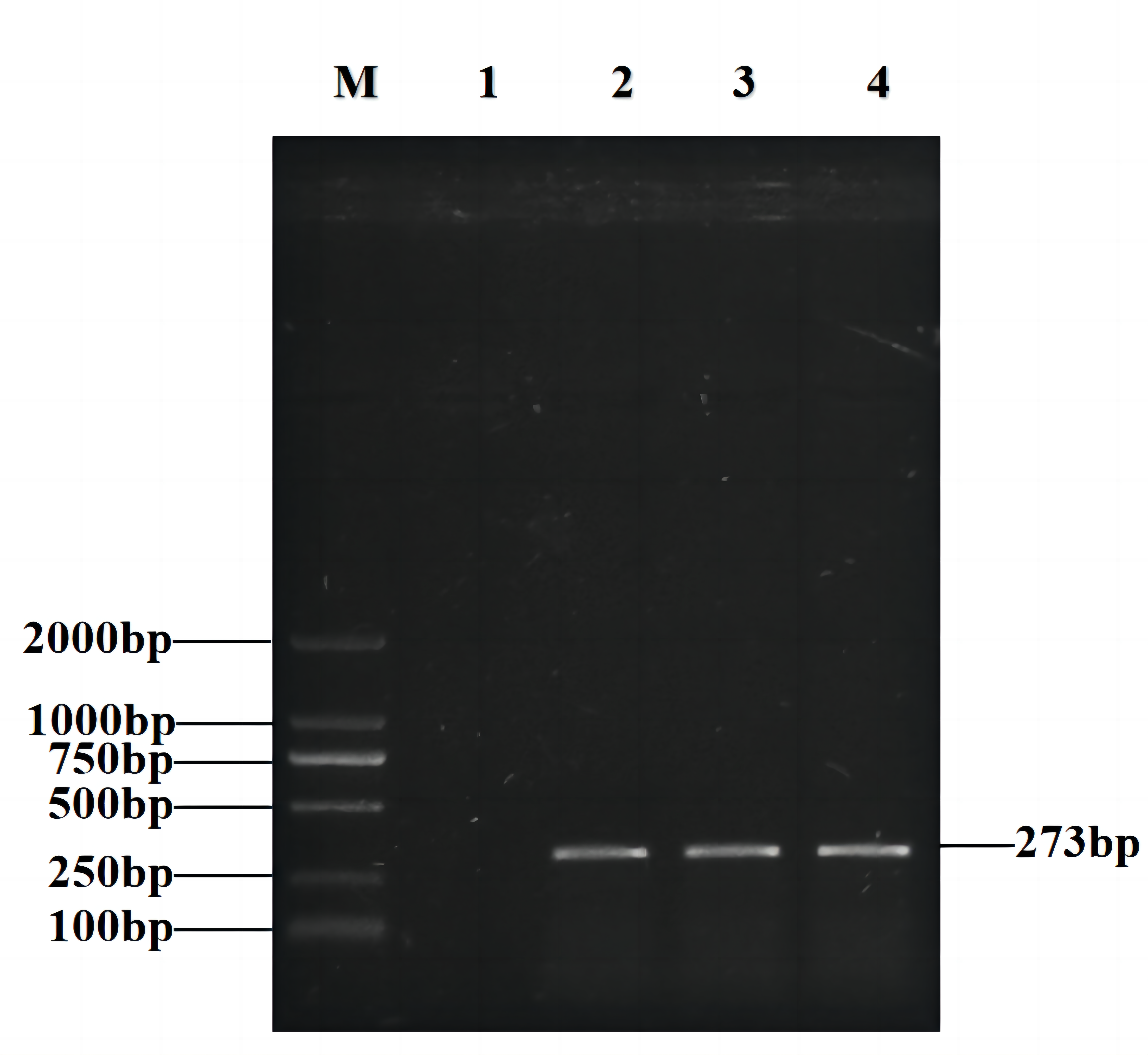

Supplement: Supplementary file 1 [file ijms-24-02751-s001.zip › Figure S5.png]
